# Assessing the Critical Thinking and Training Needs of Healthcare Professionals, and Patient Experiences: An Exploratory Cross-Sectional Study in Primary Care of Crete, Greece

**DOI:** 10.3390/healthcare14030294

**Published:** 2026-01-23

**Authors:** Antonios Christodoulakis, Anna Sergaki, Dimitrios Vavoulas, Izolde Bouloukaki, Michail Zografakis-Sfakianakis, Aristea Mavrogianni, Emmanouil K. Symvoulakis, Ioanna Tsiligianni

**Affiliations:** 1 Department of Social Medicine, School of Medicine, University of Crete, 71500 Heraklion, Greece; annasergakii@hotmail.com (A.S.); med4p1030460@med.uoc.gr (D.V.); i.bouloukaki@uoc.gr (I.B.); i.tsiligianni@uoc.gr (I.T.); 2 Department of Nursing, School of Health Sciences, Hellenic Mediterranean University, 71410 Heraklion, Greece; mzografakis@hmu.gr; 3 Department of Primary Education, School of Education, University of Crete, 74100 Rethymno, Greece; amavrog@uoc.gr; 4 Department of Primary Care and Population Health, Medical School, University of Nicosia, 2417 Nicosia, Cyprus

**Keywords:** primary care, critical thinking, training needs, patient experiences, patient satisfaction, Greece

## Abstract

**Highlights:**

**What are the main findings?**

- Primary healthcare professionals in Crete showed a high critical thinking disposition, with the highest training needs identified in research/audit, reflective practice, and initiative.
- Patients reported strong satisfaction with communication and patient-centered care, but lower satisfaction with continuity of care and empowerment, especially among those with chronic diseases.

**What are the implications of the main findings?**

- Tailored professional development programs targeting identified skill gaps can support PHC professionals in enhancing care quality.
- Policymakers should focus on improving continuity and patient empowerment through structural system changes and better care coordination.

**Abstract:**

**Background/Objectives**: Primary health care (PHC) is the cornerstone of any high-quality healthcare system. For PHC to work well, healthcare professionals need to be skilled in critical thinking, self-reflection, and patient-centered care. However, few studies have explored the potential interplays between these factors. Therefore, this cross-sectional study evaluated the critical thinking disposition and training needs of PHC professionals, alongside patient experiences and satisfaction with PHC services. **Methods**: The study involved 54 PHC professionals and 100 patients from sixteen PHC facilities in Crete, Greece. Professionals completed the Critical Thinking Disposition Scale (CTDS) and Training Needs Assessment (TNA) questionnaires, while patients filled out the Quality-of-Life Instrument of Chronic Conditions in Primary Health Care (QUALICOPC) questionnaire. **Results**: Our findings indicated that PHC professionals exhibited high critical thinking levels (CTDS, mean score of 46.46 ± 4.24). However, TNA scores suggested moderate training needs, particularly in relationships/investigations [median: 0.50 (0, 1.50)], communication/patient-centered [median: 0.30 (0, 1.1)], and flexibility and application of knowledge [median: 0.40 (0, 1.0)]. Nevertheless, no significant correlation was found between CTDS and TNA (ρ = 0.08, *p* > 0.05). Patients mostly rated their health as poor (40%), and 26% lacked a family physician. Although patients were highly satisfied with communication and patient-centered care (>95% reporting positive experiences), continuity and empowerment had room for improvement. Only 37% felt their GP knew their living conditions, and 26% lacked a personal physician. Patients with chronic conditions reported significantly different experiences. Specifically, patients with chronic conditions had better continuity of care (84% vs. 59%, *p* = 0.01) and more comprehensive care (70% vs. 43%, *p* = 0.01) compared to controls. **Conclusions**: Our findings suggest that targeted training is needed for PHC professionals to address skill gaps. These initial findings could guide the creation of customized professional development initiatives and point to areas where PHC services could be structurally improved. Additional studies, including longitudinal ones, are required to further validate these associations.

## 1. Introduction

Primary health care (PHC) is the basis of high quality healthcare systems globally, acting as the initial point of contact for patients and coordinating their care [1]. Effective PHC systems lead to lower healthcare costs, better health outcomes, and fairer access to services [2]. However, these benefits depend on a skilled and well-prepared healthcare workforce capable of delivering high-quality, patient-focused care [3,4,5]. Despite its central role, evidence shows that primary care professionals may lack key skills for optimal service delivery [3,4,5]. Studies have identified shortcomings in clinical knowledge, communication, and decision-making [3,4,5]. Beyond these skills, PHC professionals also need strong critical thinking, teamwork, and interprofessional collaboration skills [6]. These competencies are increasingly vital for navigating complex healthcare environments, thereby ensuring safe and appropriate patient care [6,7]. Addressing these skill gaps through training needs assessments is a global priority for strengthening health systems [6,7]. While many skills are needed, critical thinking stands out as especially important, even though this skill is often not as well-developed in training and understudied in practice [7].

Critical thinking skills are essential for healthcare professionals, since they are associated with better quality of care and patient satisfaction [8]. Critical thinking is defined as “a purposeful, self-regulatory judgment which results in interpretation, analysis, evaluation, and inference, as well as explanation of the evidential, conceptual, methodological, criteriological, or contextual considerations upon which judgment is based” [9]. By definition, higher levels of critical thinking in healthcare professionals could help them make better evidence-based decisions, improve the diagnostic process, adapt to the rapid changes in clinical practice, and provide safer care [8,10,11,12]. However, studies have suggested that healthcare professionals often have medium-to-low levels of critical thinking [13,14,15,16,17]. For example, a meta-analysis, incorporating studies from diverse countries such as the USA, Norway, Turkey, China, Egypt, and Taiwan, reported that nurses’ critical thinking skills generally fell in the low-to-moderate range [18]. This could lead to low quality of healthcare delivery by healthcare professionals, leading to worse patient outcomes [13,14,15,16,17]. As a result, research has emphasized the importance of developing critical thinking and other vital skills from the beginning of undergraduate studies and continuing throughout a professional’s career [19,20]. To improve the critical thinking abilities of healthcare professionals requires ongoing support and specialized educational interventions. However, the design of these interventions requires regular evaluations of training needs and assessment of patient satisfaction with healthcare services. As patient needs are constantly evolving, and the healthcare system faces ongoing challenges, it is vital to regularly assess the training needs of primary healthcare professionals to ensure that their skills stay are up to date [21,22]. These assessments could also highlight areas where further educational interventions are needed, such as in clinical judgment, communication, and leadership, thus assisting in the development of more targeted educational interventions [23]. As an example, a study conducted in Uganda indicated that primary care physicians have identified specific training needs like research, audits, and clinical work [24]. Another study from Spain has indicated that primary care nurses would benefit from additional training in communication and patient interaction [25]. All of these interventions aim to achieve patient satisfaction and patient-centered care, both critical for elevating healthcare quality and guiding healthcare reforms [26,27,28,29]. Studies further suggest that good communication (i.e., addressing patient’s concerns, giving information, following up, allowing patients to take part in decision-making, etc.) between PHC professionals and their patients can lower healthcare costs, and improve even more patient satisfaction and disease outcomes [30,31,32,33]. Therefore, evaluating training needs of PHC professionals and patient satisfaction could provide valuable insightful information to identify health systems inadequacies and improve their overall effectiveness, globally.

The inadequacies of healthcare systems were brought to light by the COVID-19 pandemic, emphasizing the fragility of health systems across the European Union, especially in Greece [34,35,36]. Specifically, the pandemic exposed the Greek health system’s inadequate preparedness, emphasizing the urgent need for improvements beyond increasing the number of workers in healthcare facilities [34,37,38,39]. These systemic pressures occurred in a system already weakened by years of economic challenges, resulting in decreased patient satisfaction and concerns about care quality [40,41]. Greece currently faces some of the highest rates of unmet medical needs in the EU, with significant disparities based on income and location [37,40,41]. Despite this urgent situation, to the best of our knowledge, there is not study in Greece that have explored the critical thinking of PHC professionals, their training needs, and patient satisfaction, simultaneously. Given that Greek health services research frequently focuses on specific aspects, such as workforce skills or patient experiences, it is challenging to understand how these aspects relate or influence each other [42,43]. Therefore, a combined assessment of these aspects could prove crucial for identifying strengths and areas for improvement within care delivery and workforce development [35,40]. In this context, this study aimed to examine the critical thinking disposition and perceived training needs of PHC professionals, while also examining patient experiences and satisfaction of PHC facilities in Crete, Greece.

## 2. Materials and Methods

This exploratory cross-sectional study followed and reported the guidelines for observational studies, specifically the STROBE guidelines [44]. A completed STROBE checklist can be found in the Supplementary Material Table S1.

### 2.1. Study Design and Sample

The study used a dual-population (healthcare providers and patients) cross-sectional approach and was conducted in sixteen (n = 16) PHC units/facilities across Crete, Greece, including facilities in the prefectures of Heraklion and Chania, from April to May 2025 (two-month period). Specifically, we included local health units (TOMY) in Heraklion (n = 4) and Chania (n = 2), plus rural Health Centers in the Chania (n = 4) area (Chania, Vamos, Kissamos) and the Heraklion (n = 6) area (Moires, Harakas, Agia Varvara, Malevizi, Archanes), which provided a mix of urban and rural PHC settings. These sixteen primary healthcare (PHC) facilities in Crete were purposefully selected to represent diverse settings. The selection considered geographical location (Heraklion and Chania prefectures), rural–urban mix (6 urban Local Health Units and 10 rural Health Centers), facility size (from small rural centers to larger urban centers), and willingness to participate. Healthcare professionals were recruited using convenience sampling. To be included, healthcare professionals had to (1) be employed at a PHC facility for at least a year to be familiar with the daily routines of the facility; (2) have direct patient care responsibilities; and (3) be willing to give informed consent. Only personnel involved in direct patient care were included, excluding support staff who did not have any contact with patients. On the other hand, patients were recruited through routine flow convenience sampling upon accepted invitation during clinic hours (8:00 to 15:00) to include a diverse group of service users. Beneficiary/patient inclusion criteria were as follows: (1) age 18 or older; (2) at least one face-to-face consultation with a frontline healthcare provider at the facility; (3) fluency in Greek; and (4) the ability to provide informed consent. Patients with major mental health disorders (at least one hospitalization) or cognitive impairments that could affect questionnaire comprehension and completion were excluded from this study.

### 2.2. Data Collection

A member of the research team visited the PHC facilities once per week for the duration of the study, during working hours, after informing the directors of each facility. Subsequently, the member approached either healthcare providers or patients and invited them to participate in the study. The member provided a detailed explanation of the study’s objectives and clarified that their responses would be anonymous (to reduce response bias) and that their participation would be voluntary. To reduce selection bias, all eligible healthcare professionals were invited to participate, and sequence sampling was used for patients. Specifically, the PHC professionals filled out the anonymous questionnaires during their breaks or at other pre-arranged times (i.e., after their shifts) to avoid interrupting patient care. Meanwhile, patients were consequently enrolled during standard healthcare units/centers hours. Patients were asked to participate right after their appointments to ensure they could easily recall their experience, and minimize any potential recall bias. The questionnaires took about 20–30 min to complete (20 for PHC professionals and about 30 min for patients). About a third of the primary healthcare (PHC) professionals agreed to participate in the study. Most declined, citing reasons like being too busy, heavy workloads, and academic fatigue. This resulted in a final study sample of 54 PHC professionals.

### 2.3. Instruments

For data collection, two anonymous questionnaires were distributed to PHC professionals the Critical Thinking Disposition Scale (CTDS) and Training Needs Assessment (TNA), and a self-report questionnaire designed for this study regarding their demographics (age, gender, job title, years of experience in primary care, current workplace, and highest level of education). Meanwhile, a self-report questionnaire for demographics that was developed for this study (age, gender, education level, current employment status, household income, household composition, and self-reported health) and two questionnaires (QUALICOPCs) were given to patients of PHC facilities to assess their satisfaction and experiences of the healthcare services. Furthermore, chronic disease status was determined by whether a patient reported having at least one chronic condition diagnosed by a doctor that needed continuous care (i.e., cancer, hypertension, diabetes, chronic respiratory diseases, cardiovascular disease, etc.).

#### 2.3.1. Critical Thinking Disposition Scale (CTDS)

The Critical Thinking Disposition Scale (CTDS), which was translated and validated in Greek, was used to measure the PHC professionals’ disposition towards critical thinking [45,46]. CTDS measures the disposition to think critically, which is considered a valid approach, since possessing the ability implies a willingness to employ it [45]. The CTDS comprises 11 statements assessing two dimensions of critical thinking: “Open-mindedness” and “Reflective Thinking”. Each query was rated on a 5-point Likert scale, with higher total scores (range 11–55) indicating a stronger disposition for critical thinking. Specifically, scores between 11 and 34 indicate low disposition; 35–44, moderate disposition; and 45–55, high disposition [45]. These two dimensions can capture the traits of a critical skeptic and reflect core elements of the definition of critical thinking. CTDS was selected for this study since it is a compact, reliable tool that examines two important aspects of critical thinking: Reflective Skepticism and Critical Openness, which makes it ideal for use in fast-paced clinical environments, including primary care settings [45]. Moreover, CTDS has also been successfully translated and consistently used in Greek populations [9,20,46].

#### 2.3.2. Training Needs Assessment (TNA)

The Training Needs Assessment (TNA) is designed to measure healthcare professionals’ professional development needs and has been translated and validated in Greek [47,48]. The TNA questionnaire comprises four sections: Section A consists of 30 items grouped into five higher-order categories: research/audit, communication and teamwork, administrative/technical, managerial/supervisory, and clinical. The first scale (A) includes evaluation queries and asks respondents to rate how important each task is to their job, producing a comprehensive professional profile score. The second scale (B) asks for a self-assessment of their current work performance, providing a skill-index score. The comparison of task importance score (A) with performance (B) produces the training needs index (A-B), where high importance coupled with low performance indicates a substantial need for training. The third scale (C) evaluates the extent to which organizational changes could improve performance on each task. The fourth scale (D) measures the extent to which proper training would enhance performance on each task. Scores for each item range from −4 to +4, where higher positive scores reflect greater training needs. To analyze at the category level, the mean score is calculated for items within each category. Scores above 0 suggest a need for training, with scores from 0.5 to 1.0 indicating moderate needs and scores above 1.0 indicating high training needs [47,48]. At the end of the questionnaire, section B comprises an open-ended question, inviting respondents to identify topics or clinical areas for further training, prioritizing those of greatest importance. Training Needs Assessment (TNA) was selected since it was designed for primary care settings, has been validated in Greek, and offers both importance and performance ratings, making it ideal for pinpointing high-priority training needs [47,48].

#### 2.3.3. Quality of Life Instrument of Chronic Conditions in Primary Health Care (QUALICOPC) for Patients’ Experiences and Patients’ Values

The QUALICOPC Patient Experiences and Patient Values Questionnaires, both translated and validated in Greek, were used for data collection [1,28]. The QUALICOPC Patient Experience Questionnaire focuses on evaluating patients’ experiences with primary healthcare [1,28]. Specifically, it comprises dichotomous (Yes/No) questions for direct feedback on specific experiences. On the other hand, the QUALICOPC Patient Values Questionnaire asked patients to rank the importance of each statement from the experience survey on a scale from 1 to 4, where 1 means not important and 4 means very important. Responses are evaluated according to the chosen scale, and the resulting scores are used to generate quality indicators such as overall satisfaction and access to healthcare. The QUALICOPC questionnaire was selected since it offers a thorough assessment of primary care, covering accessibility, continuity, coordination, comprehensiveness, and patient-centeredness [28]. This questionnaire has been widely used in European primary care studies, including studies in Greece, and facilitates comparisons across different countries [1,28].

### 2.4. Statistical Analysis

Data analysis utilized both descriptive and inferential statistics, performed with SPSS version 26.0 (IBM Corp., Armonk, NY, USA). Before inferential analyses, we checked for normality using Shapiro–Wilk tests (for n < 50) and Kolmogorov–Smirnov tests (for n ≥ 50), alongside visual inspection of Q–Q plots and histograms. The results are presented in the form of the mean ± standard deviation (SD) for continuous variables with a normal distribution, and as the median (25–75th percentile) for variables without a normal distribution. We examined the potential relationships between CTDS total scores, TNA total scores, and professional characteristics using Spearman’s correlation coefficients (since data did not meet normality assumptions, *p* > 0.05). Meanwhile, patient satisfaction data regarding primary care services was captured via the QUALICOPC questionnaire. Chi-square tests were used for categorical outcomes. Answers on the Likert scale were used to create quality indices. Patient responses to the values survey were recoded, following the method described in another study, a value of 1 indicated “very important,” while all other responses were coded as 0 [1]. We concentrated our analysis on the “very important” value category because combining positive responses (like “important” and “very important”) resulted in a ceiling effect. Mean satisfaction scores were calculated, and we used comparative tests (*t*-tests) to explore differences by patient demographics or healthcare provider characteristics. Moreover, comparisons were also made between patients with and without chronic disease, as described in another study with QUALICOPC, as experiences could differ across these patient sub-groups due to the acute care orientation of PHC services in Greece [1]. Missing data was negligible, and thus was not replaced.

## 3. Results

### 3.1. Demographics

The study involved 154 participants: 54 primary health care (PHC) professionals and 100 patients from PHC units in Crete, Greece (Table 1). Among the PHC professionals, 79.6% (n = 43) were female. Nurses made up the majority (63%, n = 34), followed by physicians (24.1%, n = 13) and other healthcare staff (12.9%, n = 7). Their average age was 42.3 ± 8.7 years (range 26–58), with 8.4 ± 5.7 years of PHC experience.

Most patients were females (57%, n = 57), with a mean age of 58.6 ± 14.2 years. Education levels were mostly moderate to high: 56% (n = 56) had post-secondary education, 30% (n = 30) had completed high school, and fewer had lower levels (Table 2).

### 3.2. Healthcare Professionals’ Critical Thinking and Training Needs

#### CTDS, TNA and Their Association

Regarding critical thinking, the overall CTDS mean score was moderate to high (mean: 46.46 ± 4.24), suggesting a generally positive approach to analytical reasoning and reflective judgment (Table 3). This was further reflected in the scores of the CTDS’s subscales; specifically, HCP professionals had a mean score of 28.74 ± 2.83 on the Critical Openness subscale and 17.72 ± 1.85 on the Reflective Skepticism subscale. The CTDS showed acceptable internal consistency, with a Cronbach’s alpha of 0.73.

The TNA indicated modest perceived educational gaps across domains (Table 3). Specifically, participants felt they needed more training in building relationships with patients [Relationships/Investigations, median: 0.50 (0, 1.50)], particularly in their communication skills [Communication/Patient-centered, median: 0.30 (0, 1.1)]. They also indicated a need for further training to more effectively bridge the gap between theory and practice [Flexibility and Application of Knowledge, median: 0.40 (0, 1.0)]. The scores indicate moderate training needs with the overall scale demonstrating good reliability (Cronbach’s α = 0.78).

No statistically significant correlation was found between CTDS total scores and TNA total scores (ρ = 0.08, *p* > 0.05) (Table 4). This suggests that the level of critical thinking and the stated training needs were not associated in this sample of PHC professionals.

### 3.3. Patient Satisfaction and Experiences of PHC Services

#### 3.3.1. Health Status and Accessibility

The 100 patients who participated in our study had considerable, unmet healthcare needs with reported poor health, and without consistent care. Specifically, from the 100, only 22% rated their health as good or very good (6% very good, 16% good), while 38% found it satisfactory, and 40% considered it poor (Table 5). A significant 43.2% (19/44) of patients without chronic conditions lacked a personal physician, contrasting with only 12.5% (7/56) of those with chronic illnesses. Within the last six months, 9% (9/100) of participants visited their GP more than five times, and all of these patients had chronic conditions. The main reasons for visiting a primary healthcare center (PHC) were feeling sick/unwell (44%) and medical check-ups (38%), with differing rates between those with and without chronic diseases: sick/unwell (51.8% with, 34.1% without) and check-up (33.9% with, 43.2% without).

#### 3.3.2. Patients’ Experiences of PHC Facilities

Table 6 presents the participants’ agreement rates with statements concerning their experiences during primary healthcare facility visits. Patients who visited frequently tend to benefit from better relationship-based and patient-centered care. On the other hand, healthier patients without chronic illnesses did not experience the same benefits of consistent care, such as a strong focus on prevention and comprehensive services that regular visits provide. Specifically, regarding accessibility, most patients considered services adequate, though nearly half reported difficulty accessing a GP outside regular hours (*p* = 0.03). Patients with chronic diseases were also more likely to visit a GP close to home or work (63% vs. 52%, *p* = 0.05). For continuity and coordination, patients with chronic conditions reported that their GP had their medical history available more often (84% chronic condition vs. 59% control, *p* = 0.01), knew important details (68% chronic condition vs. 45% control, *p* = 0.02), and was aware of their living conditions (48% chronic condition vs. 23% control, *p* = 0.01) compared to those without chronic diseases. Communication and patient-centered care received very high ratings overall (>95% for politeness, listening, and attentiveness), with no meaningful differences between groups. As regards comprehensiveness, patients with chronic disease were more likely to report that their GP could help with personal concerns (70% chronic condition vs. 43% control, *p* = 0.01) and having received preventive advice in the past year (75% chronic condition vs. 48% control, *p* = 0.01). Patient activation was high among participants, with nearly 90% reporting being included in treatment decisions, and 81% leaving the visit feeling better able to manage their health, with no significant group differences.

#### 3.3.3. Patients’ Values Questionnaire

Patients with chronic conditions seem to receive better care compared to patients without chronic conditions, including stronger relationships, improved communication, more comprehensive preparation, a holistic approach, and greater empowerment. Specifically, in terms of accessibility, patients reported very positive views, with almost all participants reporting that it was easy make appointments (99%) and felt the doctor was not under time pressure (99%) (Table 7). A notable difference was that patients with chronic disease were more likely to have telephone or email access to their doctor (86% chronic condition vs. 68% control, *p* = 0.04). For continuity and coordination, most patients believed their GP knew important details of their medical background (78%), and preparation before the consultation was more often reported by chronic patients (70% chronic condition vs. 50% control, *p* = 0.05). Communication and patient-centered care were rated very high overall, with nearly unanimous agreement that GPs listened attentively (99%) and treated patients seriously (99%). Additionally, chronic patients were more likely to feel their GP understood their personal and social background (79% chronic condition vs. 55% control, *p* = 0.01) and treated them as a person rather than just a medical problem (98% chronic condition vs. 84% control, *p* = 0.01). Regarding comprehensiveness, most patients said their GP asked about other problems (73%) and psychosocial issues (83%), though fewer received additional written information (39%) or referrals to reliable sources (28%). Patient activation was high, with 91% feeling involved in treatment decisions. Patients with chronic conditions reported greater engagement, being more likely to prepare questions (59% chronic condition vs. 36% control, *p* = 0.03), raise issues proactively (93% chronic condition vs. 73% control, *p* = 0.01), and ask questions during consultations (71% chronic condition vs. 50% control, *p* = 0.03).

## 4. Discussion

The aim of the present study was to examine the critical thinking disposition, training needs of PHC professionals, and patient experiences and satisfaction with PHC services in Crete, Greece. Our findings suggest that PHC professionals have high levels of critical thinking, with Critical Openness being particularly high. Training needs were most apparent in the areas of relationships, communication and patient-centered care, and knowledge application and flexibility. Meanwhile, patients generally expressed high satisfaction with communication and patient-centered care but reported lower satisfaction with continuity of care and patient empowerment. Interestingly, no significant correlation was found between the critical thinking abilities of PHC professionals and their self-reported training needs, suggesting these are distinct aspects of workforce development.

A major finding of the present study was that the PHC professionals of our study had high levels of critical thinking disposition. This finding aligns with other similar studies, with different measurement tools, on healthcare providers [18,49,50]. For example, a cross-sectional study of medical undergraduates found that they had moderate-to-high critical thinking levels [49]. Another study found that pediatric medical students had positive or highly positive critical thinking disposition (287.96 ± 39.09) [50]. Similarly, a meta-analysis of registered nurses found comparable levels of critical thinking levels (moderate to high) [18]. Moreover, another study among Chinese tertiary hospital healthcare workers also showed similar high critical thinking levels (281.58 ± 36.68), particularly in supportive learning environments [14]. A potential explanation for our findings could be that, while PHC professionals could have cultivated Critical Openness, perhaps due to workload pressures, they have limited time/opportunities for reflective practice (skepticism) [19,51]. Nevertheless, despite the high critical thinking levels of our participants, there is a general agreement that ongoing support and targeted educational strategies are crucial to translate these abilities into improved practice for safer and more effective care [19,51].

Our findings suggest training gaps in relationships, communication/patient-centered care, and flexibility and application of knowledge, echoing trends seen in international studies. Studies have consistently shown that healthcare professionals often have little to no formal patient communications training during their undergraduate and post-graduate training [52,53]. This often results in insufficient communication between patients and healthcare professionals [54]. Regarding flexibility and application of knowledge, healthcare professionals need to use evidence-based knowledge effectively in various clinical scenarios, yet they often lack the necessary training [55,56]. A qualitative study across four European countries has further outlined that healthcare professionals have to learn, adapt, and apply knowledge through experience, especially since their education often does not adequately cover these skills [56]. A study in a Turkish hospital also found nurses prioritizing clinical decision-making and patient teaching [57]. Moreover, a South African study of PHC professionals identified a similar need for managerial and leadership training, reflecting the complex roles within multidisciplinary teams [22]. Furthermore, a cross-sectional study on nurses’ clinical assessment experiences revealed high needs in clinical decision-making, aligning with our findings and linked to rapid technological advancements without sufficient preparation [58]. The rapid changes in PHC guidelines and patient expectations, especially in the post-pandemic era, may explain these needs, as they place considerable demands on clinicians’ practical and educational skills [59].

Another major finding of our study was that we found no statistically significant correlation between critical thinking disposition and training needs among PHC professionals, the majority of which were nurses. However, another study in clinical nurses found a positive correlation between nurses’ critical thinking disposition and their training needs [14], suggesting that healthcare professionals who think critically might be better at recognizing what they “do not” know when making clinical decisions [14]. This awareness could then drive them to proactively seek out training to fill those knowledge gaps. Moreover, this explanation also fits well with the concept of “self-directed learning” often seen in adult education [60]. Nevertheless, a potential explanation of our finding could be that critical thinking disposition reflects cognitive styles, while perceived training needs are more situational, influenced by factors like workload, role complexity, and resource availability [61]. This emphasizes the necessity of considering both internal skill improvement and external organizational factors when creating workforce development programs.

Our study also reveals high patient satisfaction with communication, access, and post-visit care, reflecting the relational strengths of Greek PHC, which aligns with national and European studies highlighting the importance of interpersonal quality, even amidst system pressures [1,62,63,64,65]. Our strong communication scores mirror the QUALICOPC findings in various countries, where patients highly valued provider explanations and saw empathy as a key factor in overcoming access difficulties [1,62,63,64,65]. Moreover, a recent study of Greek outpatients also showed similar satisfaction with politeness and attentiveness, linking this to person-centered care despite economic challenges [1]. Our findings, regarding access and satisfaction also align with a recent evaluation of urban PHC in Athens [40]. Regarding post-visit care, another study on pharmaceutical services also found high satisfaction; however, this was largely attributed to post-visit telephone support [66].

Another important finding of our study was the lower ratings for continuity of care and patient empowerment, indicating ongoing weaknesses. This is supported by other studies linking fragmented systems to patient dissatisfaction [1,62,63,64,65]. Our continuity scores agree with the results of a Spanish study, which found average management continuity among older patients, noting stronger relational ties but weaker information flow [67]. A recent review on continuity of care highlighted its link to satisfaction, while also noting issues with electronic records [68]. Similarly, patient empowerment scores align with a study where patients with chronic diseases desired greater involvement, but systemic barriers limited shared decision-making [69]. Moreover, another study showed patients prioritizing access over continuity, but our data suggests the opposite, particularly regarding after-hours care [70]. A potential explanation for our findings could be the fragmented healthcare records (no connection between hospital care and primary care records), staff turnover, and inadequate training in shared decision-making which could act as major barriers to maintaining strong patient–provider relationships and patient participation [71].

An interesting finding of this study was that patients with chronic diseases reported significantly better experiences of PHC facilities compared to those without chronic conditions, particularly in terms of continuity, coordination, and comprehensiveness of care. Patients with chronic diseases were more likely to have a personal physician, undergo frequent visits, and perceive their provider as having greater knowledge of their medical history and personal context. Furthermore, they reported feeling more supported in receiving preventive advice and psychosocial care, indicating a stronger relational dimension in primary care encounters. However, it should be noted that these benefits should be available to everyone, not just those with chronic conditions. Nevertheless, these findings are closely aligned with several European and international studies utilizing QUALICOPC and similar instruments [1,62,63,64,65]. Specifically, studies in Spain and reviews across Europe consistently report that patients with chronic conditions tend to have higher expectations for continuity and coordinated care, often rating these domains more critically when gaps exist [1,62,63,64,65]. Similar research in the Greek context highlighted satisfaction with provider communication and access, but observed lower continuity scores, especially the persistent weaknesses in system-level coordination and empowerment [1]. A plausible explanation for the observed differences lies in the fragmented nature of medical records, high staff turnover, and limited training in shared decision-making within primary care settings [72,73]. These factors undermine continuity, especially for chronic disease management where ongoing engagement and personalized care are critical. Moreover, chronic patients interact with the health system more frequently, making them more sensitive to deficits in coordination or empowerment [74].

Our findings have several implications for healthcare professionals and policymakers, highlighting the need for individual and systemic improvements to enhance PHC quality. For professionals, incorporating critical thinking into daily practice through brief reflective sessions, informed by our moderate-to-high baseline findings, can address gaps in clinical practice and teaching. Policymakers could address training needs by organizing quarterly management workshops through regional hubs, aiming to empower PHC staff by improving teamwork without adding extra workload. Policymakers could also prioritize interoperable electronic health records to improve care continuity, drawing on successful EU pilots that reduced fragmentation, and introduce incentives for longitudinal patient panels to enhance patient empowerment [68]. Furthermore, co-designing patient feedback loops using QUALICOPC metrics could inform annual audits, ensuring that strong communication sustains satisfaction while targeting empowerment through tailored training [71,75].

### Limitations

Our study, to the best of our knowledge, is the first to evaluate the critical thinking abilities and training needs of PHC professionals alongside patient satisfaction and experiences, providing valuable information for policymakers. However, despite its strengths, our study has some limitations worth mentioning. First, the exploratory cross-sectional design prevents us from establishing cause-and-effect relationships, and potentially identifying how critical thinking or satisfaction changes over time. Second, the convenience sampling (selection bias) from non-randomly selected PHC facilities of Crete limits the generalizability of the findings, particularly in rural areas. Moreover, these participants may have reported fewer training needs due to social desirability (response bias). Third, even though patients filled out the questionnaires right after their medical appointments and were told their answers would be kept confidential, we could still have been affected by social desirability or recall bias. Finally, the smaller sample size of PHC professionals restricted our ability to perform detailed sub-group analyses, and limits a geo-spatial readiness across counties of the Island of our findings. However, our sample size for this exploratory study (without funding) was considered adequate, as it was similar with other studies, for assessing PHC in Crete, Greece [76,77,78]. Nevertheless, future studies could employ longitudinal and multi-site methodologies with larger sample sizes to address these limitations. Mixed health professional group composition and convenience-driven sampling from different settings are potential reasons that may act with a confounding effect to positively relate CTDS and TNA. Since, TNA might be influenced due to the diversity of working experiences and clinical settings, a more robust sample, in terms of arithmetic composition and homogeneity, could buffer the random effects of the training needs on critical thinking interrelation. This suggestion may be useful for future studies incorporating both critical thinking and training needs assessments.

## 5. Conclusions

In conclusion, our findings suggest that PHC professionals in Crete, Greece had a high critical thinking disposition, and few training needs (communication and patient-centered care, knowledge application and flexibility, and technical/administrative procedures). Patients expressed high satisfaction with communication, access to care, and post-visit support, but highlighted that there is room for improvement in care continuity and patient empowerment. Given the exploratory cross-sectional nature of this study, the findings indicate that ongoing professional development could be improved by tailoring programs to provider needs and addressing workplace obstacles. Although these findings point to potential improvements in a relatively underexplored area of Greek primary care, future studies involving multi-centered longitudinal studies are needed.

## Figures and Tables

**Table 1:** Demographic characteristics of the healthcare professionals (n = 54).

Characteristic		N	%
Gender	Female	43	79.63
	Male	9	16.67
	Other (i.e., Gender fluid)	2	3.70
Working Position	Nurse	34	62.96
	General Practice Resident	7	12.96
	Social Worker	5	9.26
	Other	5	9.26
	Midwife	2	3.70
	General Practitioner	1	1.85

**Table 2:** Demographic characteristics of patients (n = 100).

Variables		%
Sex	Male	57
	Female	43
Were born in Greece?	Yes	82
	No	18
Were their mothers born in Greece?	Yes	68
	No	32
Other adults living in your household?	Yes	82
	No	18
Children in your household?	Yes	33
	No	67
Current employment status	Employee	36
	Freelancer/Family Business	21
	Retired	20
	Unable to work due to health conditions	13
	Household employment	8
	University Student	2
Educational Level	Junior High school	14
	High school	30
	Post-secondary	56
	(Non-Tertiary)	
Household income	Above average	10
	Around average	66
	Below average	24

**Table 3:** CTDS and TNA mean scores and mean subscale scores.

Scale and Subscales	Score
**CTDS Total**	46.46 ± 4.24
Critical Openness	28.74 ± 2.83
Reflective Skepticism	17.72 ± 1.85
**TNA Total**	0.25 (0.33, 1,4)
Communication/Patient-centered	0.30 (0, 1.1)
Research/Audit	0.29 (0, 1.21)
Flexibility and Application of Knowledge	0.40 (0, 1.0)
Technical/Administrative Procedures	0.0 (−0.06, 0.50)
Relationships/Investigations	0.50 (0, 1.50)
Reflective Practice	0.25 (0, 1.0)
Initiative	0 (0, 1.0)

Data is presented as mean values ± SD or median (25–75th percentile).

**Table 4:** Correlation matrix (Spearman’s correlation) between TNA and CTDS and their subscales.

Scales/Subscales	TNA Total Score	Communication/Patient-Centered	Research/Audit	Flexibility and Application of Knowledge	Technical/Administrative	Relationships/Investigations	Reflective Practice	Initiative	Critical Openness	Reflective Skepticism	CTDS Total Score
**TNA Total score**	1										
Communication/Patient-centered	0.86 **	1									
Research/Audit	0.78 **	0.53 **	1								
Flexibility and Application of Knowledge	0.65 **	0.50 **	0.35 **	1							
Technical/Administrative Procedures	0.56 **	0.38 **	0.25	0.46 **	1						
Relationships/Investigations	0.74 **	0.52 **	0.72 **	0.39 **	0.19	1					
Reflective Practice	0.52 **	0.24 **	0.54 **	0.27 *	0.24	0.36 **	1				
Initiative	0.55 **	0.60 **	0.31 *	0.44 **	0.32 *	0.38 **	0.15	1			
Critical Openness	0.07	0.03	0.06	0.05	−0.03	0.18	0.03	0.05	1		
Reflective Skepticism	0.06	0.08	0.03	−0.02	0.06	0.07	−0.03	0.13	0.65 **	1	
**CTDS Total Score**	0.08	0.06	0.06	0.02	0.004	0.16	−0.01	0.11	0.93 **	0.86 **	1

** *p* < 0.01; * *p* < 0.05.

**Table 5:** Participant health status, frequency of GP visits, and reason for current visit overall and by presence of a chronic disease.

Variable	Total (Ν = 100) %	Patients with Chronic Disease	Patients Without Chronic Disease	χ^2^	df	*p*-Value
		(n = 56) %	(n = 44) %			
**How would you describe your health** **status in general?**						
Very good	6 (6.0%)	1 (1.8%)	5 (11.4%)	25.41	3	<0.001
Good	16 (16.0%)	2 (3.6%)	14 (31.8%)			
Satisfactory	38 (38.0%)	21 (37.5%)	17 (38.6%)			
Poor	40 (40.0%)	32 (57.1%)	8 (18.2%)			
**Do you have your own personal physician whom you consult when you are ill?**						
Yes, the one I visited	39 (39.0%)	30 (53.6%)	9 (20.5%)	17.08	3	0.001
Yes, someone else in this facility	8 (8.0%)	3 (5.4%)	5 (11.4%)			
Yes, someone else not in this facility	27 (27.0%)	16 (28.6%)	11 (25.0%)			
No, I do not have a personal physician	26 (26.0%)	7 (12.5%)	19 (43.2%)			
**Over the past six months, how often did you visit** **or consult a General Practitioner?**						
Never	14 (14.0%)	7 (12.5%)	7 (15.9%)	14.86	4	0.005
Once	28 (28.0%)	14 (25.0%)	14 (31.8%)			
2–4 times	38 (38.0%)	24 (42.9%)	14 (31.8%)			
≥5 times	9 (9.0%)	9 (16.1%)	0 (0.0%)			
Do not know	11 (11.0%)	2 (3.6%)	9 (20.5%)			
**Reason for current visit**						
Sick/Unwell	44 (44.0%)	29 (51.8%)	15 (34.1%)	6.3	6	0.39
Medical check-up	38 (38.0%)	19 (33.9%)	19 (43.2%)			
Prescription	10 (10.0%)	5 (8.9%)	5 (11.4%)			
Referral	5 (5.0%)	3 (5.4%)	2 (4.5%)			
Health certificate	1 (1.0%)	0 (0.0%)	1 (2.3%)			
Second opinion	1 (1.0%)	0 (0.0%)	1 (2.3%)			
Other	1 (1.0%)	0 (0.0%)	1 (2.3%)			

χ^2^: Pearson chi-square test; df: degrees of freedom; GP: General Practitioner. Comparisons were performed between patients with and without chronic disease. Percentages are presented by column. Statistical significance was set at *p* < 0.05.

**Table 6:** Participants’ experience assessment by primary care domain overall and according to presence of chronic disease.

Variable	% Overall Sample (n = 100)	% Patients with Chronic Disease (n = 56)	% Patients Without Chronic Disease (n = 44)	χ^2^	df	*p*-Value
Accessibility						
Opening hours are too restricted	6	5	7	3.76	1	0.07
If I need a home visit I can get one	48	50	45	0.49	1	0.67
The practice is not close to where I live or work	57	63	52	5.45	1	0.05
I had to wait too long on the phone	38	39	38	4.26	1	0.52
The doctor took sufficient time	59	58	61	0.19	1	0.63
It was easy to make an appointment	48	45	51	0.51	1	0.67
Same- or next-day appointment	47	46	50	3.38	4	0.29
I am not able to see the GP in the evening, at night/weekend	47	50	44	4.13	1	0.03
Continuity and Coordination						
The GP had my medical history available in front of him/her	73	84	59	11.36	1	0.01
The GP knows important information about my medical history	57	68	45	5.4	1	0.02
The GP knows my living conditions	37	48	23	14.99	1	0.01
During the past two years, the GP asked me about all the medications that I was taking	83	89	77	0.51	1	0.11
It is difficult to get a referral to a medical specialist from my GP	4	2	7	0.67	1	0.21
Communication and Patient-Centered Care						
The GP was polite	98	96	100	0.61	1	0.21
The GP listened carefully to me	97	96	98	0.14	1	0.71
The GP was not looking at me when I was talking	97	97	98	0.14	1	0.71
The GP asked me questions about my health problem	98	98	98	1.6	1	0.87
I could not really understand what the GP was trying to explain to me	81	84	77	0.19	1	0.31
Comprehensiveness						
The doctor asked me about other problems beyond that for which I came	90	89	91	1.59	1	0.79
The GP is not only addressing my medical problems, but is also able to help with my personal problems and concerns	59	70	43	10.81	1	0.01
During the past 12 months, a GP talked to me about how to stay healthy (diet, smoking, alcohol)	63	75	48	6.69	1	0.01
Patient Activation						
The GP included me in the decisions about my treatment	89	89	89	0.49	1	0.92
After today’s visit, I feel that I am able to deal better with my health problem than before	81	86	75	3.45	1	0.18

χ^2^: Pearson chi-square test; df: degrees of freedom; GP: General Practitioner. Percentages are presented by column and may not total 100 due to rounding. Comparisons were performed between patients with and without chronic disease. Statistical significance was set at *p* < 0.05.

**Table 7:** Patient value survey responses by quality domain—proportion of participants who ranked as very important.

Variable	% Total (n = 100)	% Patients with Chronic Disease (n = 56)	% Without Chronic Disease (n = 44)	χ^2^	df	*p*-Value
Accessibility						
The practice has extensive opening hours	84	86	82	0.27	1	0.60
I can get an appointment easily at this practice	99	100	98	1.27	1	0.26
I know how to get evening, night, and weekend services	69	73	64	1.04	1	0.31
The practice is close to where I live or work	98	98	98	0.03	1	0.87
I have a short waiting time on the phone when I call this practice	91	89	93	0.46	1	0.50
The doctor does not give me the feeling to be under time pressure	99	100	98	1.27	1	0.26
I will keep my appointment	97	96	98	0.15	1	0.70
The doctor offers me his/her telephone or email to contact for further questions	77	86	68	4.22	1	0.04
Continuity and Coordination						
The doctor has my medical records at hand	58	63	52	1.04	1	0.31
The doctor knows important information about my medical background and health issues	78	86	70	3.47	1	0.06
The doctor knows about my living situation	50	55	45	0.94	1	0.33
I don’t need to tell a receptionist or nurse details about my health problem	38	34	43	0.87	1	0.35
The doctor has prepared for the consultation by reading my medical notes	61	70	50	3.84	1	0.05
The doctor gives me instructions on what to do when things go wrong	96	98	93	1.57	1	0.21
I inform the doctor how the treatment works out	94	95	93	0.09	1	0.76
I can see another doctor if I think it is necessary	86	86	86	0.01	1	0.93
Communication and Patient-Centered Care						
The doctor is polite	81	77	84	0.8	1	0.37
The doctor makes me feel welcome by making eye contact	96	98	93	1.56	1	0.21
The doctor listens attentively	99	100	98	1.27	1	0.26
The doctor is aware of my personal, social, and cultural background	67	79	55	6.36	1	0.01
The doctor is not prejudiced because of my age, gender, religion, or culture	95	96	95	0.06	1	0.81
The doctor treats me as a person and not as a medical problem	91	98	84	6.61	1	0.01
The doctor is respectful during physical examinations and does not interrupt me	96	98	93	1.56	1	0.21
The doctor takes me seriously	99	100	98	1.27	1	0.26
The doctor understands me	98	100	95	2.53	1	0.11
The doctor asks me if I have any questions about my health problems	95	96	93	0.52	1	0.47
The doctor asks how I prefer to be treated	65	70	59	1.17	1	0.28
The doctor avoids disturbances of the consultation by telephone calls, etc.	44	43	45	0.06	1	0.80
I understand clearly what the doctor explains	97	96	98	0.14	1	0.71
The people at the reception desk are polite and helpful	86	88	84	0.23	1	0.63
Comprehensiveness						
The doctor asks about possible other problems besides the one I came in for	73	75	70	0.25	1	0.62
The doctor gives me additional information about my health problem	39	39	39	0	1	0.95
The doctor informs me about reliable sources of information, e.g., websites	28	29	27	0.02	1	0.89
Psychosocial issues (for example personal worries) can be discussed if needed	83	82	84	0.06	1	0.80
The doctor gives all test results even if they don’t show abnormalities	87	84	89	0.43	1	0.51
Patient Activation						
The doctor involves me in making decisions about treatment	91	96	86	3.26	1	0.07
I feel able to cope better with my health problem after the visit	91	91	77	3.51	1	0.06
I have prepared for the consultation by keeping a symptom diary or preparing questions	59	59	36	4.69	1	0.03
I tell the doctor what I want to discuss in this consultation	93	93	73	6.63	1	0.01
I am prepared to ask questions and take notes	71	71	50	4.63	1	0.03
I am honest and not feeling embarrassed to talk about my health problem	100	100	95	2.53	1	0.11
I am open about my use of other treatments, such as self-medication or alternative	88	88	80	1.12	1	0.29
I adhere to the agreed treatment plan	100	100	98	1.27	1	0.26
I can bring a family member/friend to the consultation	71	71	57	2.3	1	0.13

χ^2^: Pearson chi-square test; df: degrees of freedom; GP: General Practitioner. Percentages are presented by column and may not total 100 due to rounding. All variables were dichotomous. Comparisons were performed between patients with and without chronic disease. Statistical significance was set at *p* < 0.05.

## Data Availability

The data presented in this study is available upon reasonable request from the corresponding author due to privacy restrictions.

## Supplementary Materials

The following supporting information can be downloaded at https://www.mdpi.com/article/10.3390/healthcare14030294/s1, Table S1: STROBE Statement checklist.
